# Stable Lithium Argon compounds under high pressure

**DOI:** 10.1038/srep16675

**Published:** 2015-11-19

**Authors:** Xiaofeng Li, Andreas Hermann, Feng Peng, Jian Lv, Yanchao Wang, Hui Wang, Yanming Ma

**Affiliations:** 1 Beijing Computational Science Research Center, Beijing 100084, P. R. China; 2 College of Physics and Electronic Information, Luoyang Normal College, Luoyang 471022, P. R. China; 3 Centre for Science at Extreme Conditions and SUPA, School of Physics and Astronomy, The University of Edinburgh, Edinburgh EH9 3FD, United Kingdom; 4 State Key Lab of Superhard Materials, Jilin University, Changchun 130012, Peoples Republic of China

## Abstract

High pressure can fundamentally alter the bonding patterns of chemical elements. Its effects include stimulating elements thought to be “inactive” to form unexpectedly stable compounds with unusual chemical and physical properties. Here, using an unbiased structure search method based on CALYPSO methodology and density functional total energy calculations, the phase stabilities and crystal structures of Li−Ar compounds are systematically investigated at high pressure up to 300 GPa. Two unexpected Li_*m*_Ar_*n*_ compounds (LiAr and Li_3_Ar) are predicted to be stable above 112 GPa and 119 GPa, respectively. A detailed analysis of the electronic structure of LiAr and Li_3_Ar shows that Ar in these compounds attracts electrons and thus behaves as an oxidizing agent. This is markedly different from the hitherto established chemical reactivity of Ar. Moreover, we predict that the *P4/mmm* phase of Li_3_Ar has a superconducting transition temperature of 17.6 K at 120 GPa.

Argon (Ar) is chemically quite inert due to its closed-shell electronic configuration^1^,^2^. Therefore, Ar is very reluctant to form stable compounds under ambient conditions. But ever since the first compound to contain a noble-gas atom, XePtF_6_^3^,^4^, was prepared, many scientists also speculated that other lighter noble gases should be reactive to form new compounds under suitable (most likely, low-temperature) condition^5^. Therefore, while the chemistry of Xe has developed quickly, over the last decades many scientists also tried to stabilise compounds of Argon. There are different types of Ar-containing species, including both neutral and charged molecules, such as HArF, ArH^+^, ArH_2_^+^, ArF^−^, ArCF_2_^2+^, OArF^−^, (ArO)(LiF)_2_ and so on, many of them predicted, and others observed in experiment^5^,^6^,^7^,^8^,^9^,^10^,^11^,^12^,^13^,^14^,^15^,^16^,^17^,^18^,^19^,^20^,^21^,^22^,^23^,^24^,^25^. A neutral compound involving chemically bonded Argon, hydridoargon fluoride (HArF) was isolated in a low-temperature matrix by Räsänen *et al.*^7^. Quantum-chemical calculations^7^,^8^ indicated that HArF is intrinsically stable, owing to significant ionic and covalent contributions to its bonding. This is in line with computational predictions^9^,^10^ that inferred argon should form stable hydride species. Subsequently, two new argon-containing bound metastable compounds FArCCH and FArSiF_3_ were predicted theoretically^11^. The argon compounds ArBeS^12^ and ArAuF^13^ were prepared and characterized, adding to the possible range of chemical bonds between argon atoms and other elements. Very recently, the (NgO)(LiF)_2_ (Ng = He, Ar)^17^,^18^ molecules, which contain a argon (helium) atom chemically bound to oxygen, were predicted to be stable by quantum mechanical methods. In all these compounds, whether anionic, cationic or neutral, the roles of Ar are quite different, yet all of them are weakly bound (if observable, they are only stable in low-temperature matrices) and Ar has a positive partial charge, i.e. donates electrons during the formation of the compounds. This is of particular importance for Ar chemistry, which is currently exemplified by neutral and thermally quite fragile species. Providing new physical stimuli might be a promising avenue to enrich the chemistry of argon beyond these few metastable bound compounds.

High pressure, by reducing the atomic distances and thus emphasizing the repulsive part of atomic interaction potentials, can alter the bonding characteristics of the elements, ultimately leading to novel chemical phenomena. Interactions with electropositive alkali or alkaline earth elements are particularly promising in this respect. For example, theoretical calculations of the Li-B binary system^26^,^27^ suggested that the simple stoichiometry LiB is the most stable phase under pressure, but has not yet been identified in experiment^28^,^29^. Moreover, increasing pressure makes boron (B) ions acquire more and more electronic charge, which enables B ions in Lithium-Boron compounds to transition (with increased lithium content) from graphite-like B sheets in LiB, via zigzag B chains in Li_2_B, to B_2_ dimers in Li_4_B, and finally to isolated B ions in Li_5_B and Li_6_B. In the Mg–O system, only the NaCl type structure MgO was observed in experiment at pressures up to 227 GPa^30^. However, the compounds MgO_2_ and Mg_3_O_2_ are theoretically predicted to become enthalpically stable at 116 GPa and 500 GPa, respectively^31^. The chemical bonding in both insulating MgO_2_ and semiconducting Mg_3_O_2_ exhibits significant ionic character. Dong *et al.*^32^ studied binary compounds of helium and sodium. Their results included Na_2_He, with a fluorite-type structure, and predicted to be stable above 160 GPa –well below the pressures required for compound formation of He with other elements. Other manifestations include unusual stoichiometries in the Na-Cl system^33^; stable alkali polyhydrides^34^, some with promising superconducting properties^35^; cesium in a high oxidation or an anionic state^36^; and stable Fe/Ni-Xe and Mg-Xe compounds under pressure, predicted by first principles calculations^37^,^38^. In the latter, Xe exhibits the unusual characteristic to accept electrons and form anions. The first metallic alloy of Xe, namely HgXe^6^, was in fact calculated to form at a pressure of 75 GPa, and to take up the CsCl structure. The evolution of elements’ atomic energy levels with pressure is expected to contribute to such exotic behavior^39^. Therefore, it is not unreasonable to assume that high pressure can moderate the chemical reactivity of Ar in reactions with metals. In particular, empty Ar-3*d* states are expected to become more accessible under pressure relative to the occupied *s*-states of simple metals, and Ar could serve as an electron acceptor. Lithium (Li) itself is considered to be a “simple” metal as its electronic structure is well described by a free-electron model at ambient conditions^40^,^41^. Its average electron density increases rapidly with pressure and its electronic structure changes dramatically, induced by an electronic s-p transition, eventually manifesting itself in an intermediate semiconducting phase^42^,^43^.

Here, we investigate systematically the stabilization of compounds of Li and Ar under high pressure. We employ an unbiased structure searching method as implemented in the CALYPSO (Crystal structure AnaLYsis by Particle Swarm Optimization) package^44^,^45^ in conjunction with first-principles density functional total-energy calculations to explore possible stable phases of Li-Ar system (see the Supplementary Information for more details). This method has been successfully applied to solve high-pressure structures of various systems, ranging from elements to binary and ternary compounds^35^,^36^,^37^,^38^,^43^.

## Results

Phase stabilities in the Li-Ar system are established at various pressures by judging the formation enthalpy of stoichiometric Li_*m*_Ar_*n*_ ((*m, n* )= (1,5)–(5,1); (2,3); and (3,2)) compounds. In such calculations, the formation enthalpy of per atom in Li_*m*_Ar_*n*_ is defined as follows:


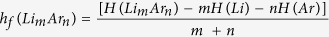


**Figure 1:**
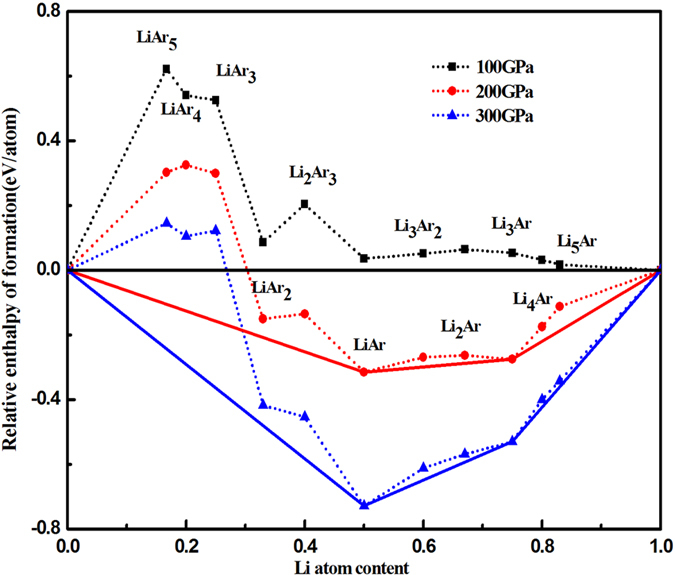
Relative enthalpies of formation per atom for Li_*m*_Ar_*n*_ phases. Dashed lines connect data points, and solid lines denote the convex hull.

There, *h*_f_ is the formation enthalpy per atom, and *H* is the calculated enthalpy of each compound. Here, we restrict ourselves to ground state calculations, i.e 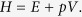

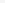
 If the formation enthalpy of a compound is negative, this compound is considered stable with respect to decomposition into the elements. For *H* (Li), we used the relevant structures across the entire pressure range^43^; for *H* (Ar), the hexagonal close packed (hcp) structure of solid Ar is adopted^46^(The enthalpies of hcp and fcc-Ar are almost equal, which does not affect the stability of the binary compounds, see Fig. S1). It is important to recognize that going beyond the ground state in light-element systems, ion dynamics can significantly change the total energies due to large zero point energy (ZPE) contributions^47^,^48^. Here, the ZPEs for the *P*4*/mmm* phase of LiAr, the hexagonal phase of Ar, and the *Cmca*-24 structure of Li at 100 GPa are calculated to be as 86, 8, and 86 meV/atom, respectively. The contribution of ZPE to *h*_f_ is thus quite small in the case of LiAr, and it is valid to neglect the contribution of ZPE when discussing the relative stability of Li−Ar systems. However, to account for all possible “escape routes”, we construct the “convex hull” or “global stability line” of all considered binary phases. In such a phase diagram, where 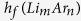 is plotted versus the lithium content 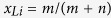, all points on the convex hull (solid line) are stable against all decomposition reactions. The convex hull for Li_*m*_Ar_*n*_ phases is displayed in Fig. 1 at 100, 200 and 300 GPa. At 100 GPa, all enthalpies of formation are positive; no Li-Ar compounds are found that are stable with respect to the elements. At 200 GPa, we see that various formation enthalpies are negative except for LiAr_*n*_ (*n* = 3, 4, 5), which indicates that other unexpected compounds Li_*m*_Ar_*n*_ are perhaps synthesizable experimentally under high-pressure conditions. Specifically, from inspecting the convex hull it is found that LiAr and Li_3_Ar are enthalpically the most stable in under high pressures. The enthalpies of other phases are only slightly higher (about 0.1 eV/atom) than those of the stable compounds. In particular, Li_4_Ar (Fig. S2, Table S1) and Li_5_Ar under high pressure, but also Li_2_Ar, Li_3_Ar_2_, LiAr_2_ and Li_2_Ar_3_ (Fig. S2, Table S1) are metastable and possibly synthesizable. More Ar-rich phases were not found to be stable at any pressure.

**Figure 2:**
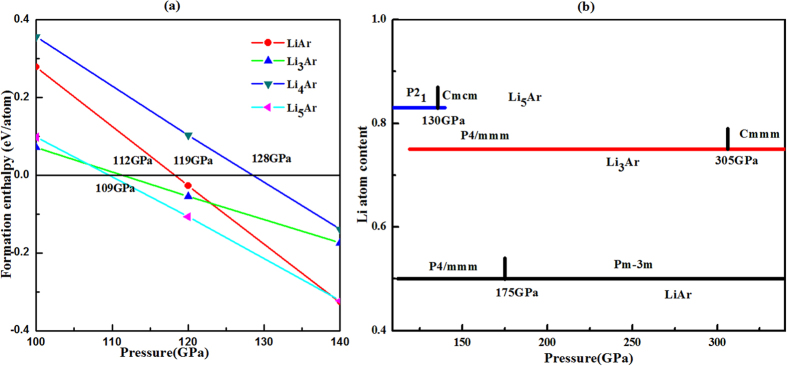
(**a**) Enthalpy of formation as a function of pressure for various Li_*m*_Ar compounds, respectively, where Δ*H*(Li_*m*_Ar) = *H*(Li_*m*_Ar)-m*H(oC_24_-Li)-*H*(hcp-Ar); (**b**) Predicted pressure ranges of stability for Li-Ar compounds.

As shown in Fig. 2, LiAr, Li_3_Ar, and Li_5_Ar become stable above 112 GPa, 119 GPa, and 109 GPa, respectively. Note that Li_5_Ar is the first Li-Ar compound stabilized by pressure, followed in quick succession by LiAr and Li_3_Ar. While Li_5_Ar is stable between about 109 GPa and 140 GPa only, both LiAr and Li_3_Ar are found to be stable up to the highest pressures included in this study (Fig. S3). In order to judge the dynamic stability of LiAr, Li_3_Ar, and Li_5_Ar, we have calculated the phonon dispersion curves (Fig. S4). No dynamic instabilities were observed throughout the whole Brillouin zone, indicating that LiAr, Li_3_Ar and Li_5_Ar are dynamically stable above 100 GPa. This means that, once formed at pressures, these phases can be decompressed down to at least 100 GPa (no dynamic instabilities were found for LiAr down to 43 GPa).

**Figure 3:**
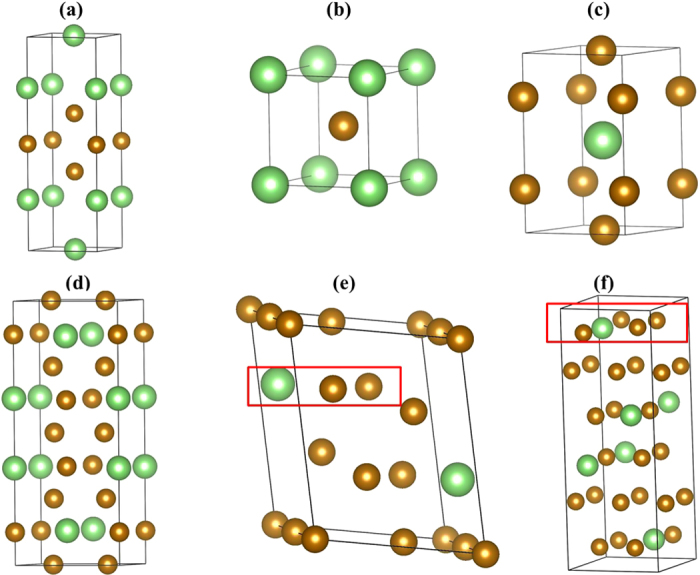
The structures of LiAr and Li_3_Ar. (**a**) *P*4*/mmm*-LiAr; (**b**) LiAr in CsCl structure type; (**c**) *P*4*/mmm*-Li_3_Ar; (**d**) *Cmmm*-Li_3_Ar; (**e**) *P*2_1_-Li_5_Ar; (**f**) *Cmcm*-Li_5_Ar. The large green balls and the smaller golden balls represent Ar atom and Li atom, respectively. Red squares indicate layers in the Li_5_Ar structures.

LiAr, Li_3_Ar and Li_5_Ar adopt relatively simple layered structures that can be interpreted as stackings of square lattices comprised of the elements (Fig. 3). Crystallographic information of the stable phases of LiAr, Li_3_Ar and Li_5_Ar are summarized in Table S2. From Fig. 2(a), it is seen that LiAr takes up a tetragonal *P*4/*mmm* phase below 175 GPa. This phase (shown in Fig. 2(a)) comprises alternately stacked square lattices of Ar and Li in [ArArArLiLiLi] periodicity along its crystallographic *c* axis. At pressures *P* > 175 GPa, the most stable structure for LiAr is the CsCl structure type, which can also be considered as alternate stackings of Li and Ar (see Fig. 3(b)). This CsCl structure of LiAr has also been found in HgXe at 75 GPa^6^. While the high-pressure phase of LiAr is one of the two most common structure type of ionic compounds (see below on a thorough examination of its electronic structure), the *P*4*/mmm* structure is not known amongst binary compounds. In fact, it can be related to the much more complex structure of the high-*T*_c_ superconductor HgBa_2_CuO_4_^49^, with lithium atoms occupying the sites of Cu and Ba cations, and some of the argon atoms occupying the sites of Hg. This comparison is not perfect, but it hints at a more complex electronic structure that stabilizes this structure for LiAr. Note that the layered nature of this structure is not an approximant to segregation. For one, *P*4*/mmm*-LiAr is more stable than the elements above 112 GPa. We also constructed larger unit cells of LiAr with [(Ar)_5_(Li)_5_] and [(Ar)_6_(Li)_6_] stacking orders; at 100 GPa, these are 0.019 eV/atom and 0.036 eV/atom higher in enthalpy than *P4/mmm*-LiAr (Fig. S5).

For Li_3_Ar, the stable structure above 119 GPa also has space group *P*4/*mmm* (stacking order [LiLiLiAr] (Fig. 3(c)). At about 305 GPa, an orthorhombic structure (space group *Cmmm*), which is also a layered structure, becomes the most stable phase of Li_3_Ar. Its conventional cell is shown in Fig. 3(d). Li_5_Ar crystallizes in the pressure range of 109 GPa to 130 GPa in a monoclinic *P*2_1_ phase, which is also a stacking variant, and shown in Fig. 3(e). Above 130 GPa, Li_5_Ar is most stable in an orthorhombic *Cmcm* phase (see Fig. 3(f)). The structures found in the stable Li-Ar phases are very similar (but not identical) to predicted Mg-Xe and Mg-Kr compounds^38^.

**Figure 4:**
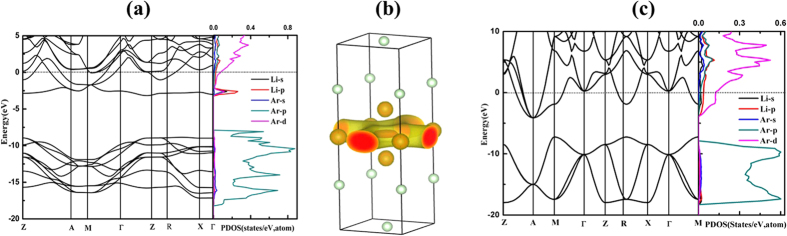
(**a**) The electronic band structure and density of states of *P*4*/mmm*-LiAr at 150 GPa; (**b**) The charge density of the flat band around −2.5 eV; (**c**) The electronic band structure and PDOS of the CsCl structure of LiAr at 200GPa.

**Figure 5:**
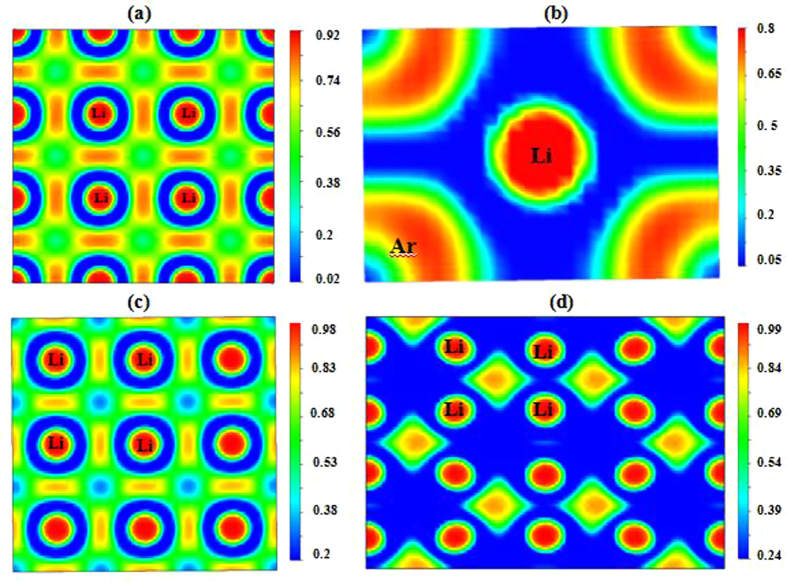
The calculated ELF of Li-Ar compounds. (**a)** *P*4*/mmm*-LiAr along (001) plane at 150 GPa, (**b**) CsCl structure of LiAr along (110) plane at 200 GPa, (**c**) *P*4*/mmm*-Li_3_Ar along (001) plane at 200 GPa, and (**d**) *Cmmm*-Li_3_Ar along (001) plane at 310 GPa. Color scheme follows the rainbow colors from blue to red.

To further investigate the nature of the stabilization of these Li-Ar compounds, we analyzed their electronic structure. Due to their predicted stability over a wide pressure range, we will focus on the LiAr and Li_3_Ar phases here. For LiAr, Fig. 4 (a) shows the electronic band structure and projected density of states (PDOS) of the *P*4*/mmm* structure at 150 GPa and the CsCl structure type at 200 GPa, respectively. Both structures are metallic, as several bands cross the respective Fermi level. The states around the Fermi level indicate a partial occupation of Ar-*3d* states in both phases, which is more pronounced in the CsCl structure at higher pressure. The same is true for the stable phases of Li_3_Ar (see Fig. S6). Moreover, we find that the density of states at the Fermi level in both Li-Ar compounds (at 150 and 200 GPa) is larger than in elemental Li at the same pressures (Fig. S7). Note that *P*4*/mmm-*LiAr has a very flat band at 2 eV below the Fermi energy. We plot the charge density of this band in Fig. 4(b), and find the electron density to be partially localized in the interstitial region in the Li layer. Li itself undergoes a structural phase transition at about 70 GPa and becomes a semiconducting ‘electride’^50^,^51^, a phenomenon that is caused by localization of valence electrons in the interstitial region of a densely packed Li lattice^42^. In contrast, tetragonal LiAr remains metallic under high pressure throughout its stability range. This is caused by partial charge transfer from Li-2*s* to Ar-3*d* states. A topological analysis of the electron density based on Bader’s atoms-in-molecules approach^52^ helps us to quantify this effect: the net atomic charges in *P*4*/mmm*-LiAr at *P* = 160 GPa are + 0.71e for Li1, + 0.59e for Li2, −0.12e for Ar1 and −0.40e for Ar2. Note that these charges do not add up to zero: Bader’s analysis finds two pockets of electronic charge in the interstitial region, at the *2e* (1/2, 0, 1/2) position in the unit cell, with −0.54e in each pocket. These interstitial electrons are at the same position as oxygen anions in the CuO_2_ layers of HgBa_2_CuO_4_. Because the lithium atoms’ valence electrons are not completely localized, but also partially populate the Ar-3*d* states, the structure remains metallic, in contrast to the pure lithium “electride” phase. The localized nature of the interstitial electron is confirmed by the Electron Localization Function (ELF), which carries information about the bonding character and valence electron configurations of atoms in a compound^53^. Larger ELF values usually correspond to inner shell or lone pair electrons and covalent bonds, whereas ionic and metallic bonds correspond to small ELF values. In Fig. 5(a), ELF is shown for a cut through the *z* = 1/2 plane of tetragonal LiAr at 150 GPa: besides the 1 s^2^ shell of lithium, there is significant interstitial electron localization visible between adjacent lithium cations (with a maximum value of ELF = 0.87).

The CsCl phase of LiAr at 200 GPa is also found to be metallic and from Fig. 4(c), we can see that the value of DOS at the Fermi level is larger than that of tetragonal LiAr at 150 GPa. This indicates that the charge transfer is larger in this structure (and at the higher pressure), as Ar-3*d* states are lowered relative to Li-2*s* states. Bader’s analysis gives partial charges of + 0.67e for Li and −0.67e for Ar at *P* = 200 GPa, which reduce to ± 0.62e at *P* = 300 GPa. As could be expected, no interstitial charge density forms in this structure – see the plot of the ELF in the [110] plane at *P* = 200 GPa in Fig. 5(b). Thus, at pressures above *P* = 175 GPa, LiAr is predicted to form a simple intermetallic compound – with anionic character of Ar. The Li core does not contain p-orbitals and is thus quite compact, due to the absence of orthogonality constraints, which contributes to the stability of LiAr with respect to elemental Li and Ar.

For Li_3_Ar, the electronic band structure and PDOS of the tetragonal *P*4*/mmm* phase at 200 GPa and the orthorhombic *Cmmm* phase at 310 GPa are compiled in Fig. S4. They indicate that Li_3_Ar under high pressure is metallic in either phase. As in LiAr, this is driven by partial occupation of the Ar-3*d* states. The ELF plots for Li_3_Ar at 200 GPa and 310 GPa (see Fig. 5(c,d)) show that, similar to tetragonal LiAr, there is electron localization in the interstitial region between Li atoms. A Bader analysis corroborates this picture: in tetragonal Li_3_Ar, the partial charge of Li1 is + 0.61e at 200 GPa (+0.57e at 300 GPa), + 0.55e (+0.49e) for Li2, and −1.03e (−1.02e) for Ar. The interstitial site at *2f* (1/2, 0, 0) has a partial charge of −0.46e (−0.37e). In the orthorhombic structure of Li_3_Ar at 310 GPa, the charge transfer numbers agree qualitatively with all other Li-Ar phases discussed here.

Since all predicted stable phases are metallic, we estimated their potential for phonon-mediated superconductivity, with electron-phonon coupling calculated with the Quantum ESPRESSO package^54^. In our calculations we found that neither of the stable LiAr high-pressure phases exhibits significant electron-phonon coupling. However, for the tetragonal *P*4*/mmm*-Li_3_Ar, we find an electron-phonon coupling strength of λ = 0.721 and a superconducting temperature *T*_c_ = 17.6 K at 120 GPa, which reduce to λ = 0.454 and *T*_c_ = 6.5 K at 200 GPa. Superconductivity in an electride has been measured at ambient pressure: in 12CaO·7Al_2_O_3_, a superconducting phase was found below *T*_*c*_ = 0.4 K^55^.

In summary, by crystal structure searches based on CALYPSO methodology and density functional total energy calculations, potentially stable Li−Ar phases are systematically investigated at high pressure up to 300 GPa. Two unexpected Li_*m*_Ar compounds (LiAr and Li_3_Ar) might be experimentally synthesizable over a wide range of pressures. Our calculations indicate that LiAr and Li_3_Ar are enthalpically and dynamically stable above pressures of 112 GPa and 119 GPa, respectively, while Li_5_Ar is stable in a small pressure window of 109–140 GPa. We found that all stable phases are metallic.

High pressure can induce argon to become an electron acceptor, as evidenced here by its ability to form stable intermetallic compounds with Li. In this particular system, the formation of ionic compounds (involving charge transfer from Li-2 s to Ar-3d states) competes with lithium’s propensity to shed its valence electron into interstitial space. The first stable Li-Ar compounds are thus predicted to feature both electride and metallic behavior. With higher pressure, the tendency of electron localization decreases in favor of increased ionic character. The absolute value of electronic charge transferred gradually decreased for all stable Li-Ar compounds. Amongst the stable Li-Ar compounds, Li_3_Ar exhibits reasonable electron-phonon coupling, with predicted superconducting temperatures of 17.6 K at 120 GPa and 6.5 K at 200 GPa, thus adding this compound to an intriguingly short list of candidates of superconducting electride phases.

## Computational Methods

The crystal structural predictions were performed by CALYPSO methodology as implemented in the CALYPSO code^43^,^44^. The significant feature of this methodology is its capability of predicting stable and metastable structures at given pressures with only the knowledge of the chemical composition. The structures of Li_*m*_Ar_*n*_ (*(m, n)* = (1,5)-(5,1); (2,3); and (3,2)) were investigated at pressures of 100, 200 and 300 GPa. The underlying ab initio structural relaxations and electronic band structure calculations used density functional theory with the Perdew-Burke-Ernzerhof (PBE) generalized gradient approximation (GGA) of the exchange-correlation energy as implemented in the VASP code^56^. The projector augmented wave (PAW) method was used to model the electron-ion interaction^56^, including the 1 s^2^2 s^1^, and 3 s^2^3p^6^ electrons of Li and Ar, respectively, in the valence space. A cutoff energy of 900 eV was used for the plane wave expansion of the wave functions, and fine regular k-point grids used for Brillouin zone integrations^57^, to ensure that all the enthalpy calculations were converged to better than 1 meV/atom. The accuracy of the total energies obtained within the framework of density functional theory is in many cases sufficient to predict the stability of structures. Phonon calculations were carried out using a supercell approach as implemented in the PHONOPY code^58^. Estimates of electron-phonon coupling strengths and phonon-mediated superconductivity were obtained using the Quantum ESPRESSO code^54^; see the Supplementary Information for more details.

## Additional Information

**How to cite this article**: Li, X. *et al.* Stable Lithium Argon compounds under high pressure. *Sci. Rep.* **5**, 16675; doi: 10.1038/srep16675 (2015).

## Supplementary Material

Supplementary Information
