# Supplementary material for: Stable Lithium Argon compounds under high pressure
Source: Sci Rep. 2015 Nov 19;5:16675. doi: 10.1038/srep16675 (PMC4652216; doi:10.1038/srep16675)
Supplement: Supplementary Information [file srep16675-s1.pdf]

Supporting information

Stable Lithium Argon compounds under high pressure

Xiaofeng Li [1, 2], Andreas Hermann [3], Feng Peng [2], Jian Lv [1], Yanchao Wang [4], Hui Wang [4], Yanming Ma[1, 4, *],

1Beijing Computational Science Research Center Beijing, 100084, P. R. China

2College of Physics and Electronic Information, Luoyang Normal College, Henan Luoyang, 471022, PR China

3 Centre for Science at Extreme Conditions and SUPA, School of Physics and Astronomy, The University of Edinburgh, Edinburgh EH9 3FD, United Kingdom

4 State Key Lab of Superhard Materials, Jilin University, Changchun 130012, Peoples Republic of China

[*]Email of corresponding author: mym@calypso.cn ; webpage: http://mym.calypso.cn

1. Computational details

We employ a global minimization of free energy surfaces based on ab initio density functional (DFT) total-energy calculations and a particle swarm optimization (PSO) algorithm as implemented in the CALYPSO code. Structure searches were performed at 100, 200 and 300GPa with up to four formula units for Li_mAr_n ((*m*,*n*)=(1,5)-(5,1); (2,3); and (3;2)). In each search, in the first step, random structures of random space group symmetry are constructed in which the atomic coordinates are generated by the crystallographic symmetry operations. Then the structures are optimized to local minima by using DFT (VASP code) calculations. After processing the first generation, 60% of the structures, those with the lowest enthalpies, are selected to produce the next generation structures by PSO. 40% of the structures in the new generation are randomly generated. A structure fingerprinting technique of bond characterization matrix is applied to the generated structures, so that identical structures are strictly forbidden. These procedures significantly enhance the diversity of the structures, which is crucial for the efficiency of the global search of structures. The local optimizations are performed by use of the conjugate gradient method and the criteria of the enthalpy change is 2×10^{-5}eV per atom. In most cases, the structure searches reach convergence after 50 generations covering about 2500 structures. Note that all stable phases bar Li_3Ar-*Cmmm* contain less than four f.u. in the unit cell. In order to ensure all possible relevant crystal structures for the LiAr composition were located, we also searched for structures of LiAr with simulation cell sizes of 6 f.u., 8 f.u and 10 f.u. at pressures of 100, 200 and 300 GPa. No new structures emerged. However, while one can not exclude the possibility that more complex structures are even more stable than those found here, structure searches with larger unit cells were not computationally feasible for other compositions.

2. Superconducting temperature

We calculated the superconducting transition temperature T_c of the stable Li-Ar compounds using the Allen–Dynes form of the McMillan[1] equation:

$$T_c = \frac{\omega_{\ln}}{1.2}\exp\left[-\frac{1.04(1+\lambda)}{\lambda-\mu^*(1+0.62\lambda)}\right]$$

There, $\lambda(=2\int_0^\infty \alpha^2F(\omega)/\omega d\omega)$ is the electron-phonon coupling constant, $\omega_{\ln}$ is the logarithmic average phonon frequency, and μ^* is the Coulomb pseudopotential. The logarithmic average frequency was calculated by

$$\omega_{\ln} = \exp \frac{2}{\lambda} \int_0^{\infty} \frac{\alpha^2 F(\omega) \ln \omega}{w} d\omega$$

The Eliashberg spectral function, $\alpha^2 F(\omega)$, which measures the contribution of the phonons with frequency ω to the scattering of electrons, can be written as [2]

$$\alpha^2 F(\omega) = \frac{1}{2\pi N(\varepsilon_F)} \sum_{qv} \frac{\gamma_{qv}}{\omega_{qv}} \delta(\omega - \omega_{qv})$$

Here, $N(\varepsilon_F)$ is the electronic DOS at the Fermi level. The line width of each phonon mode was calculated as

$$\gamma_{qv} = 2\pi\omega_{qv} \sum_{kjj'} \left| g^{qv}_{k-qj',kj} \right|^2 \delta(\varepsilon_{kj} - \varepsilon_F) \delta(\varepsilon_{k+qj'} - \varepsilon_F)$$

Where $g^{v}_{k-qj',kj}$ is the electron-phonon coupling matrix element. The Coulomb pesudopotential μ^* was given the typical value 0.10 in our calculation.

Supplemental Figures

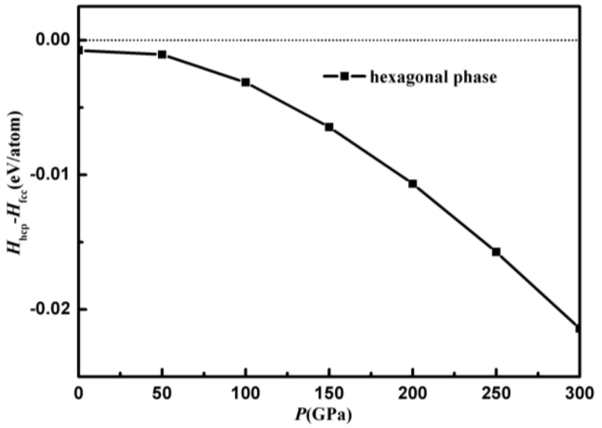

Figure S1 The enthalpy differences of hexagonal and fcc phase of solid Ar under pressure

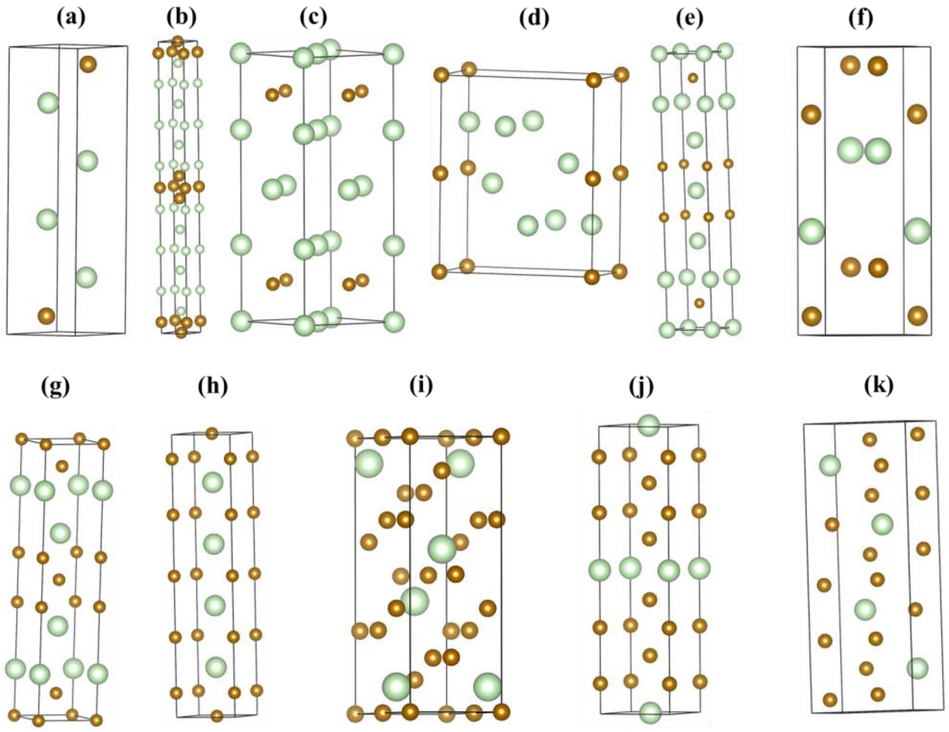

Figure S2 $LiAr_2$: (a) P-3M1 phase at 100GPa; (b) I4/mmm phase at 200GPa; (c) P4/mmm phase at 300GPa. Li_2Ar_3: (d) Pmma phase at 100GPa; (e) P4/mmm phase at 200GPa and 300GPa. Li_2Ar: (f) P4/nmm phase in the whole pressure range. Li_3Ar_2: (g) I4/mmm phase at 100GPa; (h) P4/mmm phase at 200GPa and 300GPa for. Li_4Ar: (i) Cmcm phase at 100GPa; (j) I4/mmm phase at 200GPa; (k) Pnma phase at 300GPa.

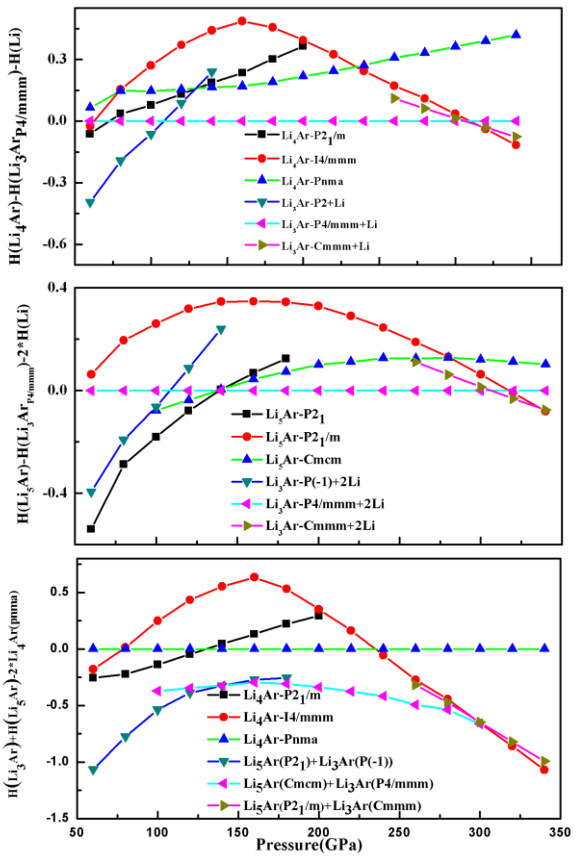

Figure S3 The relation of enthalpy of Li-Ar compounds with pressure.

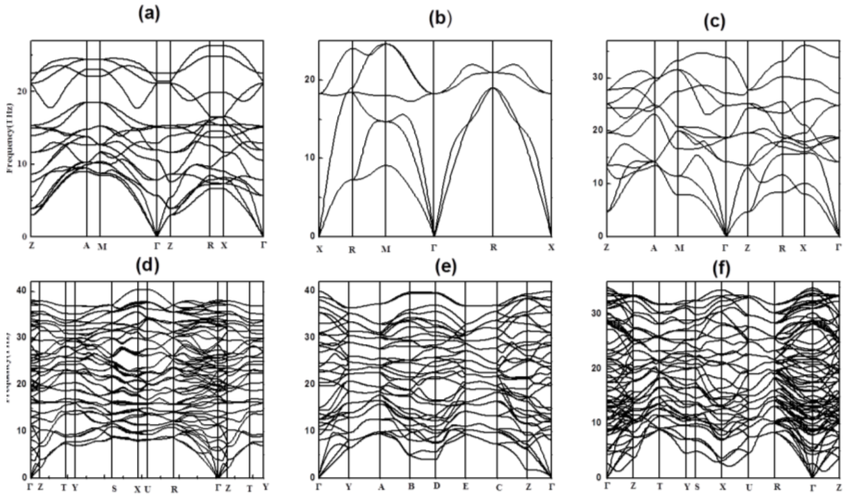

Figure S4 Phonon dispersion curves for (a) tetragonal P4/mmm-LiAr at 120GPa, (b) Pm-3m-LiAr at 200GPa, (c) tetragonal P4/mmm-Li_3Ar at 120GPa, (d) orthorhombic Cmmm-Li_3Ar at 320GPa,(e) $P2_1$-Li_5Ar at 110GPa, and (f) Cmcm-Li_5Ar at 140GPa.

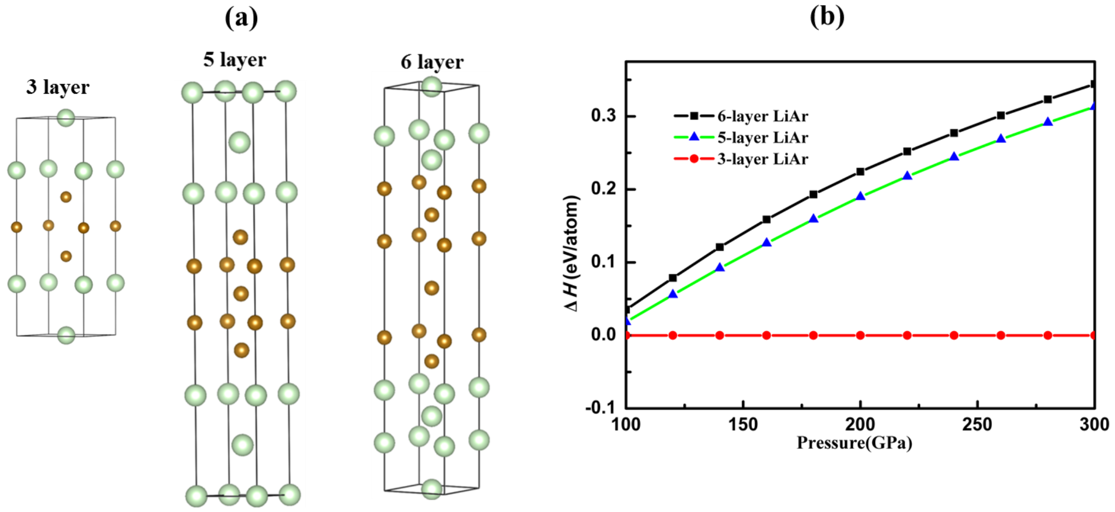

Figure S5 (a) the LiAr with 3 layer, 5-layer and 6-layer [Ar, Li] stacking. (b)The relative enthalpies of LiAr structures including 5-layer and 6-layer [Ar,Li] stacking, compared to the P4/mmm phase LiAr with 3-layer [Ar,Li] stacking.

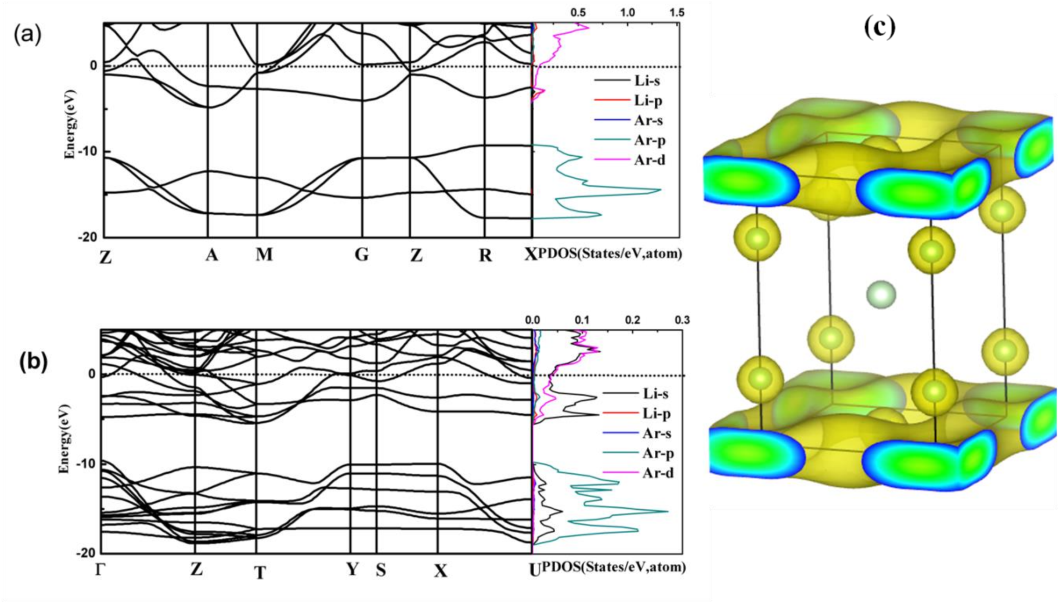

Figure S6 The electronic band structure and PDOS of Li_3Ar (a) tetragonal P4/mmm structure at 200GPa (b) orthorhombic Cmmm structure at 310GPa.(c) The charge density between -5eV and -2.5eV below the Fermi level in the P4/mmm phase.

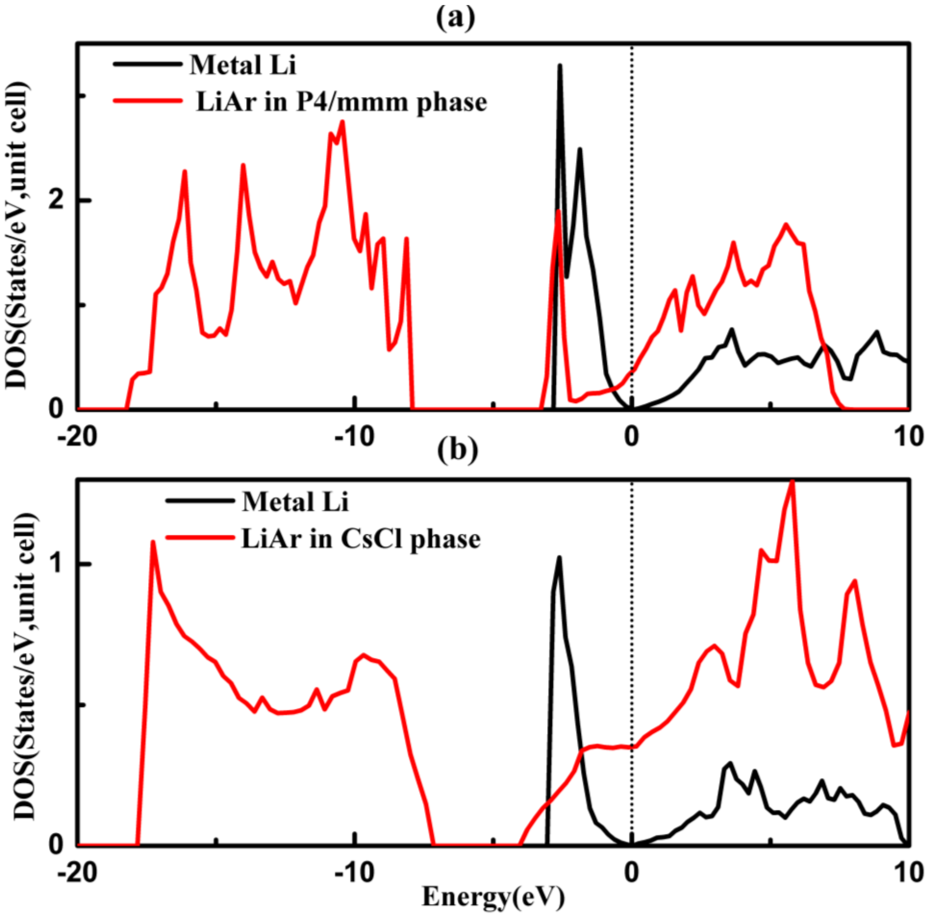

Figure S7 The total density of states of LiAr and metal Li (a) 150GPa, Li is Cmca-24 phase (b) 200GPa, Li is Cmca-56 phase.

Supplemental Tables

Lattice parameters	atoms	x	y	z
$LiAr_2$				
P-3M1 at 100GPa				
a=*b*=2.597Å	Li1 (2d)	0.66667	0.33333	0.06231
c=10.586Å	Ar1 (2d)	0.66667	0.33333	0.80426
	Ar2 (2d)	0.66667	0.33333	0.39865
*I*4/mmm at 200GPa				
a=*b* =2.430 Å	Li1(4e)	-0.00000	0.00000	0.03859
c=24.672Å	Li2(2b)	0.50000	0.50000	-0.00000
	Ar1(4e)	0.50000	0.50000	0.35502
	Ar2(4e)	0.50000	0.50000	0.21494
	Ar3(4e)	0.50000	0.50000	0.92578
*P*4/mmm at 300GPa				
a=*b*=2.276 Å	Li1(2h)	-0.50000	-0.50000	-0.14702
c=7.910 Å	Ar1(2g)	0.00000	0.00000	-0.28499
	Ar2(1a)	0.00000	0.00000	0.00000
	Ar4(1d)	-0.50000	-0.50000	-0.50000
Li_2Ar_3				
*P*mma at 100GPa				
a=6.306Å	Li1(2a)	0.00000	0.00000	0.00000
b=2.797Å	Li2(2f)	0.25000	0.50000	0.90178
c=5.401Å	Ar2(2e)	0.75000	0.00000	0.44241
	Ar2(4j)	0.54950	0.50000	0.75229
*P*4/mmm at 200GPa				
a=*b*=2.358 Å	Li1(4e)	-0.00000	-0.00000	0.59331
c=13.138 Å	Ar1(4e)	-0.00000	-0.00000	0.81964
	Ar2(2a)	0.00000	0.00000	0.00000
Li_2Ar				
*P*4/nmm at 200GPa				
a=*b*=2.431 Å	Li1(2c)	0.50000	0.00000	0.06510
c=6.191 Å	Li2(2c)	0.00000	0.50000	0.22635
	Ar1(2c)	0.50000	0.00000	0.35614
Li_3Ar_2				
*I*4/mmm at 100GPa				

a=*b*=2.598 Å	Li1(2a)	0.00000	0.00000	0.00000
c=11.901 Å	Li2(4e)	0.50000	0.50000	0.91266
	Ar1(4e)	0.50000	0.50000	0.67029
*P*4/mmm at 200GPa				
a=*b*=2.378 Å	Li1(2g)	0.00000	0.00000	0.08664
c=10.818 Å	Li2(2g)	0.00000	0.00000	0.28456
	Li3(1b)	0.00000	0.00000	0.50000
	Li4(1c)	0.50000	0.50000	0.00000
	Ar1(2h)	0.50000	0.50000	0.60862
	Ar2(2h)	0.50000	0.50000	0.17356
Li_4Ar				
*C*mcm at 100GPa				
a=2.571Å	Li1(8f)	0.50000	0.19929	1.08335
b=7.606Å	Li2(4c)	0.50000	0.12201	0.75000
c=7.264Å	Li3(4a)	0.00000	0.00000	0.50000
	Ar1(4c)	0.00000	0.90722	0.75000
*I*4/mmm at 200GPa				
a=*b*=2.405 Å	Li1(4e)	0.00000	0.00000	0.69984
c=9.170 Å	Li2(4e)	0.50000	0.50000	0.39428
	Ar1(2b)	-0.00000	-0.00000	0.50000
*P*nma at 300GPa				
a=3.649Å	Li1(4c)	0.91901	0.25000	0.44204
b=2.529Å	Li2(4c)	0.82293	0.25000	0.65061
c=9.530Å	Li3(4c)	0.92459	0.25000	0.24911
	Li4(4c)	0.39191	0.25000	0.45522
	Ar1(4c)	0.32762	0.25000	0.64558

Table S1. Lattice parameters and atomic positions of metastable Li-Ar compounds

Lattice parameters	atoms	x	y	z
LiAr				
*P*4/mmm at 160GPa	Li1 (2h)	0.5000	0.5000	0.6350
a=*b*=2.491Å	Li2 (1b)	0.0000	0.0000	0.5000
c=7.295Å	Ar1 (1c)	0.5000	0.5000	0.0000
	Ar2 (2g)	0.0000	0.0000	0.7579
*P*m-3m at 260GPa	Li1 (1a)	0.5000	0.5000	0.5000
a=*b*=*c*=2.297 Å	Ar1(1a)	0.0000	0.0000	0.0000
Li_3Ar	Li1 (1a)	0.0000	0.0000	0.7571
*P*4/mmm at 200GPa	Li2 (1a)	0.0000	0.0000	0.2429
a=*b*=2.406 Å	Li3 (1a)	0.5000	0.5000	0.0000
c=3.768 Å	Ar1 (1a)	0.5000	0.5000	0.5000
*C*mmm at 310GPa				
a=3.625Å	Li1 (4i)	0.0000	0.8745	0.0000
b=8.579Å	Li2 (4h)	0.2408	0.0000	0.5000
c=2.373Å	Li3 (4f)	0.2500	0.2500	0.5000
	Ar1(4i)	0.5000	0.1315	0.0000
Li_5Ar				
$P2_1$ at 120GPa				
a=4.099Å	Li1 (2e)	0.7314	0.7500	0.2996
b=3.778Å	Li1 (2e)	0.5939	0.7500	0.6994
c=5.056Å	Li1 (2e)	0.7304	0.2500	0.6274
$\alpha = \gamma = 90^{\circ}, \beta = 103.1^{\circ}$	Li1 (2e)	0.5798	0.2500	0.0101
	Li1 (2a)	0	1.0000	1.0000
	Ar1(2e)	0.0574	0.7500	0.6744
Cmcm at 140GPa				
a=3.855Å	Li1(8d)	0.2500	0.7500	0.5000
b=9.924Å	Li2(4c)	0	0.0922	1.2500
c=3.724Å	Li3(8g)	0.2456	0.9228	1.2500
	Ar(4c)	0.5000	0.9035	0.7500

Table S2. Lattice parameters and atomic positions of LiAr, Li_3Ar and Li_5Ar

References

1. McMillan, W. L. Transition Temperature of Strong-Coupled Superconductors. *Phys. Rev.*, **167**, 331-332 (1968).

2. Eliashberg, G. M. Interactions between electrons and lattice vibrations in a superconductor. *Sov. Phys.-JETP*. **11**, 696 (1960).
